# Chromophobe renal cell carcinoma mimicking cortical invasion by synchronous invasive urothelial carcinoma of the intra-renal collecting system on CT urography

**DOI:** 10.1590/S1677-5538.IBJU.2014.0596

**Published:** 2016

**Authors:** Ryan Yu, Gabriella Gohla, Ehsan A. Haider

**Affiliations:** 1 Department of Pathology and Molecular Medicine, McMaster University, Hamilton, Ontario, Canada; 2 St. Joseph's Hospital, Hamilton, Ontario, Canada; 3 Department of Diagnostic Imaging, McMaster University, St. Joseph's Hospital, Hamilton, Ontario, Canada

## CASE PRESENTATION

**Figure 1:**
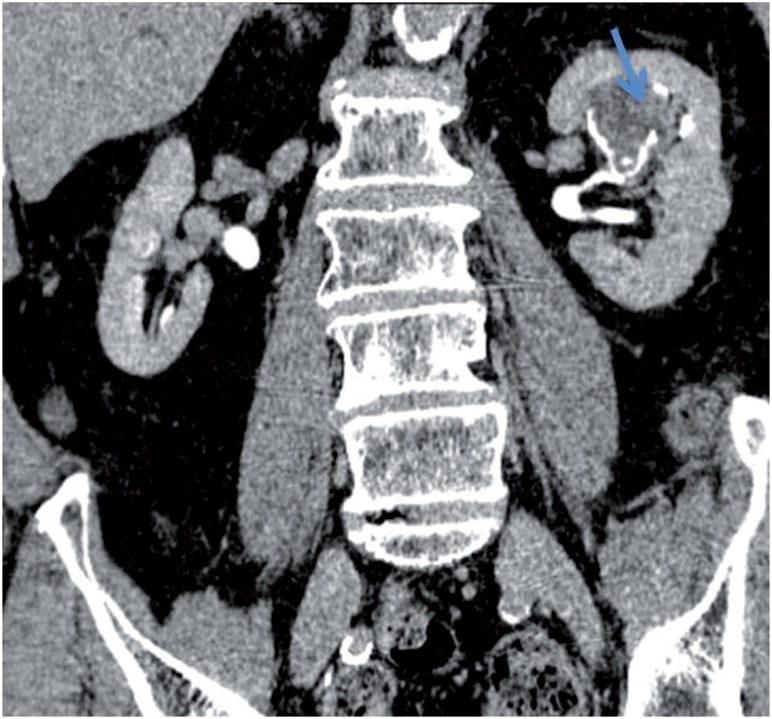
Coronal 20 minute excretory urogram shows a lobulated soft tissue mass (urothelial carcinoma) casting the left kidney upper pole calices (straight arrow).

**Figure 2:**
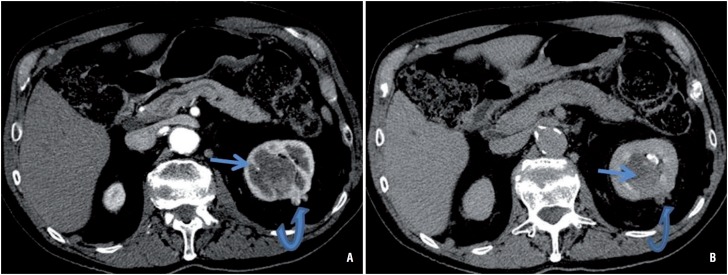
A) Axial corticomedullary phase shows a less heterogeneously enhancing urothelial carcinoma associated with parenchymal invasion of the upper pole of the left kidney (straight arrow) and the subjacent more enhancing exophytic renal cell carcinoma (curved arrow); B) Excretory urogram shows the intra-renal collecting urothelial carcinoma and its parenchyma invasion (straight arrow) are of similar attenuation to the renal cell carcinoma nodule (curved arrow).

**Figure 3:**
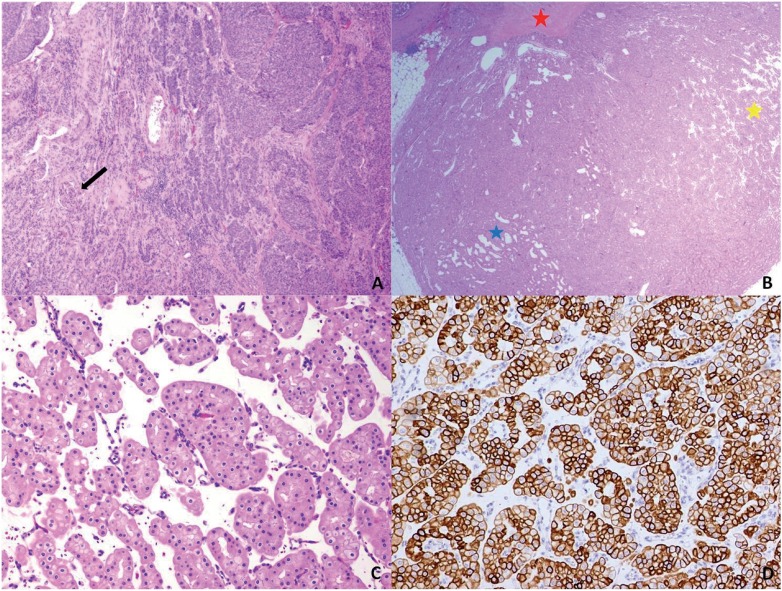
A) Urothelial carcinoma invading to the renal cortex (H&E, 40x). Note the glomerulus (arrow); B) Deceptive oncocytoma-like architecture with archipelagic nests (yellow star), tubules (blue star), and fibrous scar (red star) (H&E, 12.5x); C) The nests are composed of tumor cells with well-defined cell membranes, perinuclear clearing, and non-uniform nuclei (H&E, 100x); D) Cytokeratin 7 shows strong, diffuse immunostaining of the tumor cells at the cell membrane (100x).

An 83-year-old man presented to hospital with gross hematuria. His medical history was remarkable for remote open left pyelolithotomy, chronic inflammatory demyelinating peripheral neuropathy, benign prostatic hyperplasia, and cerebrovascular accident for which he took aspirin-dipyridamole. Urine cytology was negative for malignant cells. Renal ultrasound showed a lobulated, hypoechoic lesion centered in an echogenic hilum of the upper pole of the left kidney. Preoperative CT urogram showed a large, round, heterogeneously-enhancing soft tissue filling defect, measuring 3.6cmx4.0cmx3.3cm, casting the major calyx of the upper pole of the left kidney (Figure-1). It showed definitive heterogeneous enhancement after contrast administration. On the delayed urographic phase, contrast was noted outlining the filling defect. The findings were typical for urothelial carcinoma, with invasion of the subjacent parenchyma of the upper pole of the left kidney. In addition, an irregular, exophytic 1.4cmx1.4cm nodule was found arising from the posterolateral cortex of the left kidney, in the vicinity of the renal parenchymal invasion by the urothelial carcinoma. The nodule was homogeneously hyper-enhancing and appeared contiguous with the less enhancing urothelial carcinoma (Figures 2A and B). The nodule was thought to be either urothelial carcinoma invasion or a synchronous renal cell carcinoma. There was no local regional lymphadenopathy. He underwent laparoscopic nephroureterectomy without complication. Pathologic examination confirmed urothelial carcinoma invading the renal parenchyma to the corticomedullary junction and an adjacent chromophobe renal cell carcinoma with a pushing border into perinephric fat (Figures 3A-D).

## DISCUSSION

Urothelial carcinoma of the renal pelvis accounts for 12% of all renal tumors, while renal cell carcinoma accounts for 85% (1). The synchronous occurrence of urothelial carcinoma and renal cell carcinoma in the same kidney is exceedingly rare (2). Reported patients are usually men who present with hematuria at a mean age of 65 years (3). In this case, it is likely that the invasive urothelial carcinoma was the cause of the patient's hematuria, while the renal cell carcinoma was an incidental lesion. Preoperative radiologic delineation of urothelial carcinoma and renal cell carcinoma in the same kidney is usually straightforward when they are geographically distinct. However, they may be found in very close proximity (4). For accurate preoperative interpretation, the urologist should be aware that the juxtaposition of urothelial carcinoma that has invaded the renal cortex with a cortically-limited chromophobe renal cell carcinoma may present as an apparently single lesion on CT urography. In this case, the difference in contrast enhancement in the early phase supported the presence of two distinct lesions, despite apparent contiguity given their same attenuation on the delayed phase. The prognosis for patients with two malignancies is likely most influenced by the malignancy with the highest stage.
